# The Effect of Dietary Glycaemic Index on Glycaemia in Patients with Type 2 Diabetes: A Systematic Review and Meta-Analysis of Randomized Controlled Trials

**DOI:** 10.3390/nu10030373

**Published:** 2018-03-19

**Authors:** Omorogieva Ojo, Osarhumwese Osaretin Ojo, Fajemisin Adebowale, Xiao-Hua Wang

**Affiliations:** 1 Department of Adult Nursing and Paramedic Science, University of Greenwich, London SE9 2UG, UK; 2 Healthcare, Care UK, HMP Wormwood Scrubs, London W12 0AE, UK; Osarhumwese.Ojo@careuk.com; 3 Department of Animal Production and Health, Federal University of Technology, PMB, Akure 704, Ondo State, Nigeria; debofajemisin@yahoo.co.uk; 4 The School of Nursing, Soochow University, Suzhou 215006, China; wangxiaohua@suda.edu.cn

**Keywords:** glycaemic index, glycated haemoglobin, fasting blood glucose, type 2 diabetes, randomised controlled trials, meta-analysis, systematic review

## Abstract

Background: The increasing prevalence of diabetes in the United Kingdom and worldwide calls for new approaches to its management, and diets with low glycaemic index have been proposed as a useful means for managing glucose response. However, there are conflicting reports and differences in the results of studies in terms of their effectiveness. Furthermore, the impact of low-glycaemic index diets and their long-term use in patients with type 2 diabetes remains unclear. Objectives: The objective of this study was to conduct a systematic review and meta-analysis of the effect of low-glycaemic index diets in patients with type 2 diabetes. Methods: Search methods: Randomised controlled studies were selected from a number of databases (EBSCOHost with links to Health Research databases, PubMed, and grey literature) based on the Population, Intervention, Comparator, Outcomes and Study designs (PICOS) framework. The search terms included synonyms and Medical Subject Headings (MeSH) and involved the use of Boolean operators (AND/OR) which allowed the combination of words and search terms. Selection criteria: As per the selection criteria, the following types of articles were selected: studies on randomised controlled trials, with year of publication between 2008 and 2018, including patients with type 2 diabetes. Thus, studies involving patients with gestational and type 1 diabetes were excluded, as were observational studies. Nine articles which met the inclusion criteria were selected for the systematic review, whereas only six articles which met the criteria were included in the meta-analysis. Data collection and analysis: Studies were evaluated for quality and risk of bias. In addition, heterogeneity, meta-analysis, and sensitivity tests of the extracted data were carried out using Review Manager 5.3 (Review Manager, 2014). Results: The findings of the systematic review showed that the low-glycaemic index (low-GI) diet resulted in a significant improvement (<0.05) in glycated haemoglobin (HbA1c) in two studies: low-GI diet Δ = −0.5% (95% CI, −0.61% to −0.39%) vs. high-cereal fibre diet Δ = −0.18% (95% CI, −0.29% to −0.07%); and low-GI legume diet Δ = −0.5% (95%, −0.6% to −0.4%) vs. high-wheat fibre diet Δ = −0.3% (95% Cl, −0.4 to −0.2%). There was a slight improvement in one study (low glycaemic response = 6.5% (6.3–7.1) vs. control = 6.6% (6.3–7.0) and no significant difference (*p* > 0.05) in four studies compared with the control diet. Four studies showed improvements in fasting blood glucose in low-GI diets compared to higher-GI diets or control: low-GI diet = 150.8 ± 8.7 vs. higher-GI diet = 157.8 ± 10.4 mg/dL, mean ± SD *p* = 0.43; low-GI diet = 127.7 vs. high-cereal fibre diet = 136.8 mg/dL, *p* = 0.02; low-GI diet = 6.5 (5.6–8.4) vs. standard diabetic diet = 6.7 (6.1–7.5) mmol/L, median and interquartile range *p* > 0.05; and low-GI diet = 7.3 ± 0.3 vs. conventional carbohydrate exchange diet = 7.7 ± 0.4 mmol/L, mean ± SEM (Standard Error of Mean) *p* < 0.05. The results of the meta-analysis and sensitivity tests demonstrated significant differences (*p* < 0.001 and *p* < 0.001, respectively) between the low-GI diet and the higher-GI diet or control diet in relation to glycated haemoglobin. Differences between the low-GI diet and higher-GI diet or control were significant (*p* < 0.05) with respect to the fasting blood glucose following meta-analysis. Conclusion: The low-GI diet is more effective in controlling glycated haemoglobin and fasting blood glucose compared with a higher-GI diet or control in patients with type 2 diabetes.

## 1. Introduction

The increasing prevalence of diabetes and its impact on morbidity and mortality have become global problems [1,2]. About 422 million adults worldwide were reported to live with diabetes in 2016, and the global prevalence rose from 4.7% in 1980 to 8.5% in 2014 [1]. In the United Kingdom, the prevalence of type 2 diabetes more than doubled from 2.39% in the year 2000 to 5.32% in 2013 [2]. The management of type 2 diabetes and its related complications, including retinopathy, kidney dysfunction, neuropathy, and foot problems accounts for about 10% of the entire National Health Service (NHS) budget in the UK [2]. Presently, 11% of U.S. adult population has diabetes and the total estimated costs associated with the condition in 2012 were US $245 billion due to direct medical costs and reduced worker productivity [3]. Several factors, including genetic predisposition and environmental factors, have been implicated in the aetiology of diabetes [3,4]. This is particularly true in the case of type 2 diabetes, which accounts for over 90% of all forms of diabetes and where lifestyle has a profound effect on its manifestation [5]. Usually, lifestyle factors such as diet and physical activities can be modified in terms of the choices that individuals make. The composition of diet with respect to the quality of the nutrients including carbohydrates, protein, fats, minerals and vitamins is important in determining nutritive value and usefulness in human health [6].

### 1.1. Description of the Intervention 

Foods that are composed of carbohydrates which break down quickly during the process of digestion (such as white bread) and that are rapidly absorbed into the blood stream are often termed as foods with high glycaemic index (GI) [7,8,9]. Foods with high GI not only rapidly increase blood glucose, but also insulin responses following the consumption of food [10]. In contrast, foods with a low glycaemic index such as legumes, lentils, and oats usually contain carbohydrates which break down slowly during digestion and are slowly assimilated [8,9]. Therefore, these foods have a slower impact on blood glucose levels and insulin response. 

The GI is a measure of the percentage of the area under curve (AUC) with respect to 2-h blood glucose following the ingestion of a test diet compared with a standard diet (usually glucose or bread) [7]. It can also be viewed as a reflection of the relative rate of digestibility of the available carbohydrates of the food compared with a reference food, which is often glucose [11,12]. Differences exist in literature as to what constitutes a low-GI diet and a high-GI diet. Values such as GI ≤ 40 and GI ≤ 55 for the low-GI diet and GI ≥ 70 for the high-GI diet have been reported [7,13]. 

### 1.2. How the Intervention Might Work 

The GI value of food is not based on the characteristics of the individual that consumed it, instead, it depends on the food consumed [9,14,15]. Therefore, dietary management approaches which target weight loss and improved glycaemic control (including glycated haemoglobin and fasting blood glucose) in patients with type 2 diabetes may rely on the use of diets with low glycaemic index instead of using standard low-fat diet [16]. The foods with low GI may contribute to glycaemic control compared to foods with high GI through the promotion of insulin sensitivity, reducing fluctuations in blood glucose levels and reducing daily insulin requirements [8]. While glycated haemoglobin (HbA1C) provides a measure of the average glycaemia over the preceding 3 months, the fasting blood glucose is a measure of blood glucose level following at least 8 h of fasting, and is usually taken before breakfast [17]. 

### 1.3. Why It Is Important to Do This Review 

Strategies for managing diabetes often rely on lifestyle modifications, including dietary interventions and pharmacological approaches. There is also evidence that the consumption of diets with high glycaemic index and glycaemic load over a long period of time may have implications for metabolism and health, including chronic hyperglycaemia and hyperinsulinaemia, which can lead to insulin resistance and diabetes [14]. In addition, studies involving populations in China and the USA have shown that women with a high intake of food with a high glycaemic index were more at risk of developing type 2 diabetes compared with women on diets with low glycaemic index [14,18,19]. However, there are inconsistencies and controversies with respect to the use of GI of food as a guide in the selection of foods for patients with diabetes [8,12,20,21,22]. Evidence from previous studies on the role of diets with low GI on health and health-related outcomes have produced mixed results [16]. While some studies have found the high-GI diet to be related to poorer short-term metabolic outcomes, greater hunger, less satiety, and greater food intake [10], the results from other studies have been different, either not finding the same association or finding an inverse relationship [12,23]. Jung and Choi [13] demonstrated the beneficial effects of low-GI diet on glucose control in relatively short-term trials in patients with type 2 diabetes, although the long-term effects of low-GI diets remain unclear. This view is further reinforced by Thomas and Elliott [8] who noted that the effects of low-GI diets in managing patients with diabetes have demonstrated mixed results, from small but clinically useful effects on the medium-term glycaemic control in diabetes, to only modest secondary benefit. The review by Thomas and Elliott [8], which was published more than 7 years ago and involved patients with type 1 and type 2 diabetes, found that there was a significant decrease in HbA1c in a low-GI diet compared with control. Some of the studies included in this review involved children, and the primary outcome measures were HbA1c and fructosamine. Another review on glycaemic index and type 2 diabetes included only observational studies [22].

However, the current systematic review is based only on randomised controlled trials and involves only adults with type 2 diabetes, and the outcomes of interest are HbA1c and fasting blood glucose. In addition, there is currently no globally agreed form of diet for managing patients with diabetes [8]. Therefore, research on how best to understand the quality and composition of carbohydrates and other nutrients in foods will be essential in developing diets that will one day be useful to patients with diabetes and acceptable to the global community.

### 1.4. Objectives 

This is a systematic review and meta-analysis which evaluates the effect of the low-glycaemic index diet in patients with type 2 diabetes

Research question: Is a low-GI diet effective in improving glycaemia in patients with type 2 diabetes compared with a higher-GI diet?

## 2. Methods 

### 2.1. Types of Studies 

Only studies involving randomised controlled trials were selected for this review (Table 1).

### 2.2. Types of Participants 

The participants in the studies selected were adult patients with type 2 diabetes (Table 2).

### 2.3. Types of Interventions 

The effect of low-glycaemic index diet was compared with higher-glycaemic index diet or control (conventional carbohydrate exchange, high-cereal fibre diet, high-wheat fibre diet, standard diabetic diet, American diabetes association diet) in adult patients with type 2 diabetes. The higher-GI or control diets were classified as having a higher glycaemic index based on the lower GI values of the intervention diets (low-GI diet).

### 2.4. Types of Outcome Measures 

The following were the outcome measures of interest:

Blood glucose parameters: Glycated haemoglobin (%), fasting blood glucose (mg/dL).

#### Search Methods for Identification of Studies

The Population, Intervention, Comparator, Outcomes and Study designs (PICOS) framework was used to identify articles in the various databases [24,25]. The search terms included synonyms and Medical Subject Headings (MeSH) and involved the use of Boolean operators (AND/OR) which allowed the combination of words and search terms (Table 1).

### 2.5. Electronic Searches 

**Figure 1:**
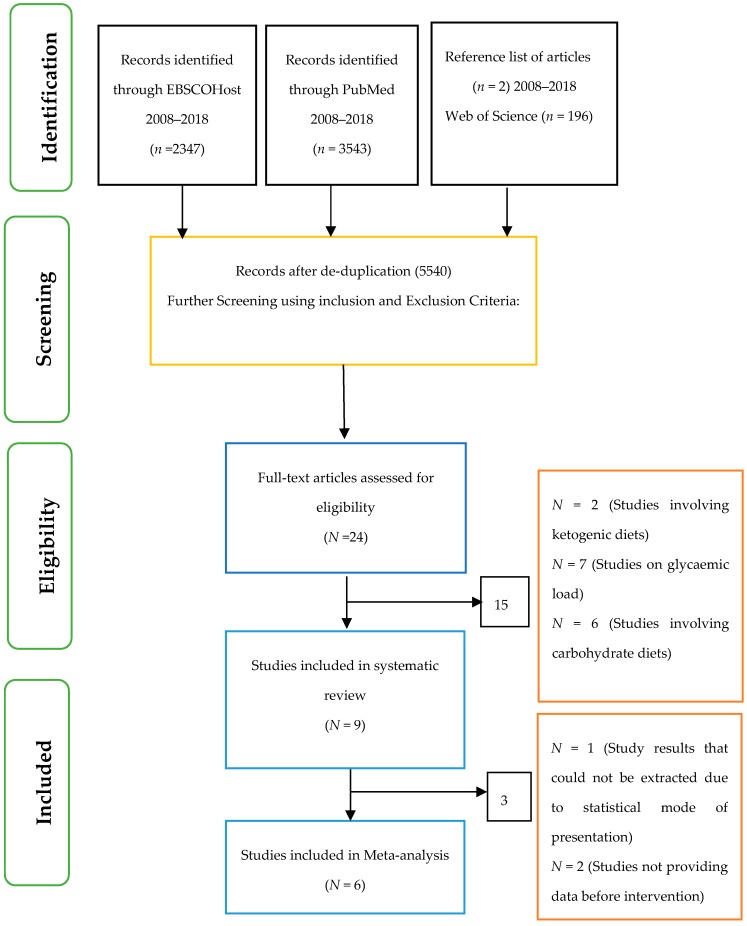
PRISMA flow chart showing the selection of articles.

A number of research databases were used to search for relevant articles for this review. These included EBSCoHost research databases with links to Health Research databases which incorporate Academic Search Premier, Medline, the Psychology and Behavioural Sciences Collection, PSYCInfo, and the Cumulative Index to Nursing and Allied Health Literature (CINAHL) Plus. In addition, Pubmed was searched for useful articles (Figure 1).

### 2.6. Searching Other Resources 

The Web of Science database which encompasses the BIOSIS citation index was searched for conference papers, and the reference list of articles were also searched.

### 2.7. Selection of Studies 

Only primary research on randomised controlled studies carried out between 2008 and 2018 were included in this review (Table 2). This period was chosen because the search period for the previous systematic review and meta-analysis by Thomas and Elliot study [8] ended in March 2009. Earlier search conducted from 2009 to 2018 did not yield enough studies for the current review.

In addition, only studies involving adults with type 2 diabetes and the use of the dietary glycaemic index were included. Studies written in English from across the world have been included as diabetes is a worldwide problem.

Therefore, other studies involving patients with type 1 diabetes or gestational diabetes and animal studies were excluded from this review (Table 2). Similarly, studies involving children with diabetes or healthy adults without diabetes were also excluded. Studies which were not randomised and those involving dietary supplements have been excluded from this review.

### 2.8. Evaluation of Quality

The quality of the peer-reviewed articles was evaluated using the checklists for quantitative studies [26] and the experience of the authors.

### 2.9. Data Extraction and Management 

Data from the selected articles were extracted separately by all the authors based on an agreed framework and verified by all the authors following completion. 

### 2.10. Meta-Analysis Methods

The meta-analysis of data was carried out using Review Manager (RevMan) 5.3 software [24]. Changes in means and standard deviations between the baseline values and final results for each outcome of interest in the low-GI diet and the higher-GI diet or control for the different studies were determined. In addition, the number of participants in the intervention and control groups in each study were included in a table and entered into the RevMan software for analysis. A heterogeneity test was carried out in order to evaluate the evidence of variability of the intervention effects [27]. For the heterogeneity test, a *p* value of 0.1 was used to determine statistical significance [27]. The heterogeneity statistic *I*^2^ value was ˂50 and this indicated low heterogeneity for the studies included in both the glycated haemoglobin and fasting blood glucose analyses. Therefore, the fixed effects model was used for both the meta-analysis and the sensitivity tests.

A fail-safe number was also calculated for both outcomes of interest. A sensitivity analysis involving a repeat of the meta-analysis of studies that were definitely known to be eligible was also conducted [27]. This process involved removing some of the studies from the primary analysis in order establish that the findings from the systematic review were not dependent on unclear decisions [27]. In this case, the sensitivity tests were carried out for both glycated haemoglobin and fasting blood glucose by repeating the meta-analysis after removing the studies with the most weight in order to confirm whether the results were stable. 

### 2.11. Assessment of Risk of Bias in Included Studies 

The assessment tool used for evaluating the risk of bias was a domain-based evaluation tool [27]. The process involved the separate critical assessment of the various domains including the random sequence generation (selection bias), allocation concealment (selection bias), blinding of participants and personnel (performance bias), blinding of outcome assessment (detection bias), incomplete outcome data (attrition bias), selective reporting (reporting bias), and other bias [27]. The risk of bias was assessed by Review Manager 5.3 software [24]. 

## 3. Results 

With respect to the systematic review, only nine articles [28,29,30,31,32,33,34,35,36] met the criteria for inclusion (Table 3). However, six of these studies [28,29,30,31,35,36] were used for the meta-analysis to test the effect of low-GI diet on glycated haemoglobin and fasting blood glucose in patients with type 2 diabetes. The others were excluded as they did not meet the criteria for meta-analysis such as reporting their results in the form of median and interquartile or not providing data before intervention.

The length of study ranged from 2 weeks to 22 months. In terms of the interventions, these involved comparing the low-GI diet with the higher-GI diet or control (conventional carbohydrate exchange, high-cereal fibre diet, high-wheat fibre diet, standard diabetic diet, American diabetes association diet) (Table 3). 

In most of the studies [28,30,34,35,36] that reported dietary glycaemic index values, the low-GI diet had significantly (*p* < 0.05) lower values than the higher-GI diet or the control. The study by Jenkins et al. [29] also showed that the low-GI diet resulted in lower GI values than the high-cereal fibre diet, although the level of statistical difference was not stated (Table 3). However, differences in dietary GI values were not significant (*p* > 0.05) in one study [31], while data were not available in two of the studies [32,33] although the authors stated that the intervention diets involved low glycaemic index or low glycaemic response.

In studies reporting on the effect of dietary GI on the HbA1c levels, diets with low GI were shown to result in a significant improvement (*p* < 0.05) in HbA1c levels in two studies [29,30] compared with the higher-GI diet or the control. One study [33] reported slight improvement in HbA1c in the low-GI diet group compared with control, while differences between low-GI diet and higher-GI diet or control were not significantly different in four studies [31,34,35,36]. 

The effect of low-GI diets on fasting blood glucose compared to higher-GI diets or control diets was evident in seven of the studies selected [28,29,30,32,33,34,36]. While four studies [28,29,34,36] showed a greater improvement in fasting blood glucose in the low-GI diet compared with higher-GI diet, some of the differences were not statistically significant. Furthermore, there was a lower fasting blood glucose level in the higher-GI diet or control compared with low-GI diet in two studies [30,33]. The fasting blood glucose levels were not significantly different in the low-GI diet compared with control in one other study [32].

### 3.1. Assessment of Risk of Bias in Included Studies 

**Figure 2:**
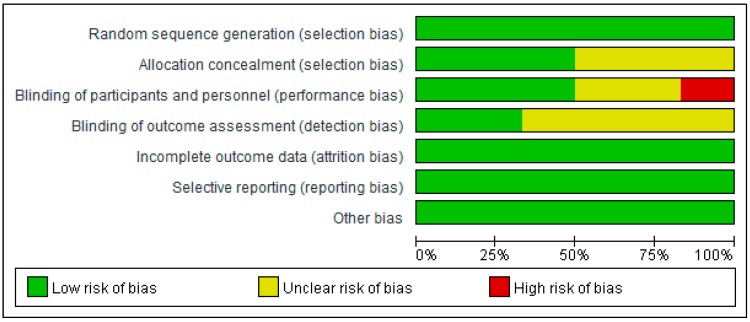
A risk of bias summary.

**Figure 3:**
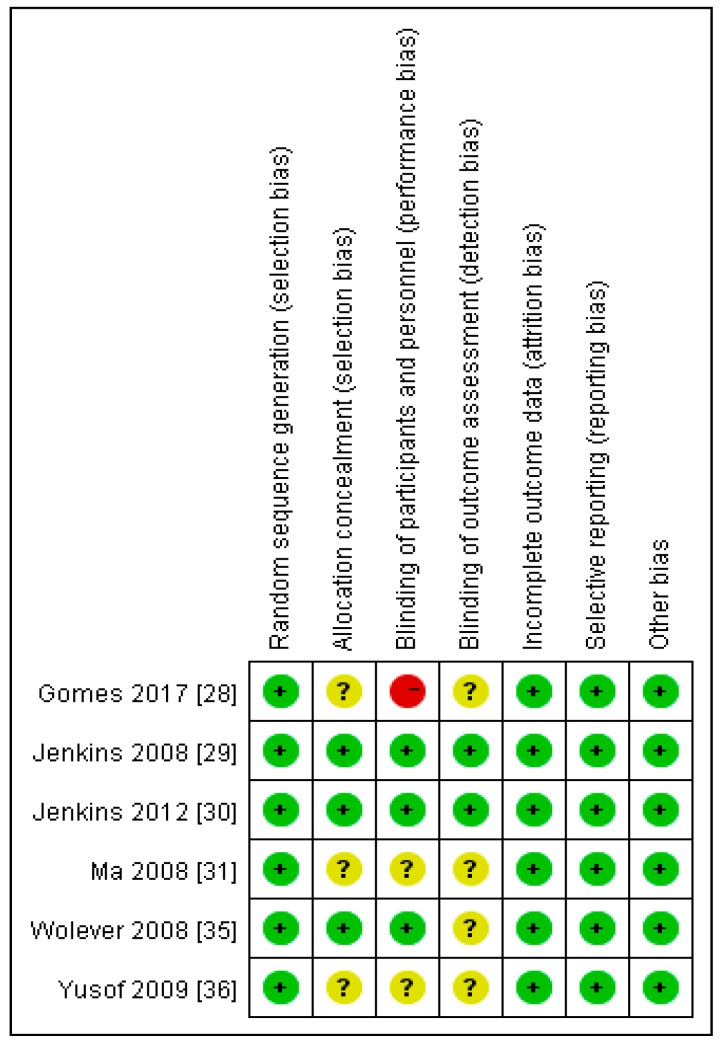
A risk of bias graph.

In terms of the selection bias (random sequence generation and allocation concealment) 100% of the studies showed low risk of bias (Figure 2 and Figure 3). With respect to the other risks of bias (blinding, incomplete outcome data, selective reporting and other potential sources of bias), all the studies demonstrated 100% low risk of bias or unclear risk of bias (Figure 2 and Figure 3) except for the Gomes et al. study [17] which demonstrated high risk of bias in relation to the blinding of participants and personnel. 

### 3.2. Effect of Low Glycaemic Index on Glycated Haemoglobin

**Figure 4:**
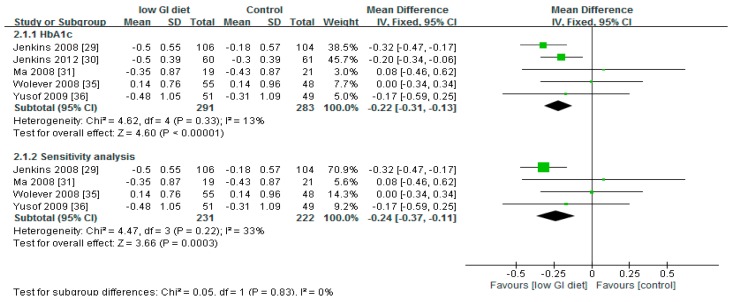
A forest plot showing the effect of low-GI diet on glycated haemoglobin (%).

The test of the overall effect of the low-GI diet compared to higher-GI diet or control on HbA1c showed that the low-GI diet was more effective both in the meta-analysis (*p* < 0.001) and in the sensitivity test (*p* < 0.001) (removing the study with the most weight) (Figure 4). 

There was low heterogeneity (*p* = 0.33) in the studies used to evaluate the effect of low-GI diet on glycated haemoglobin. The *I*^2^ test showed *I*^2^ = 13% for meta-analysis and *I*^2^ = 33% for the sensitivity test, again, confirming low heterogeneity of the studies included.

The result of the sensitivity test (Figure 4) showed that the effect of the low-GI diet on glycated haemoglobin was reliable. According to *N*_fs0.05_ = (∑Z/1.64)^2^ − S (Z representing the Z value of each single study; S representing the number of all enrolled studies) to calculate the fail-safe number (*N*_fs0.05_)^24^, the *N*_fs0.05_ was 16. That is, another 16 negative studies would be needed to reverse this result, thus indicating that the result is stable.

### 3.3. Effect of Low Glycaemic Index on Fasting Blood Glucose

**Figure 5:**
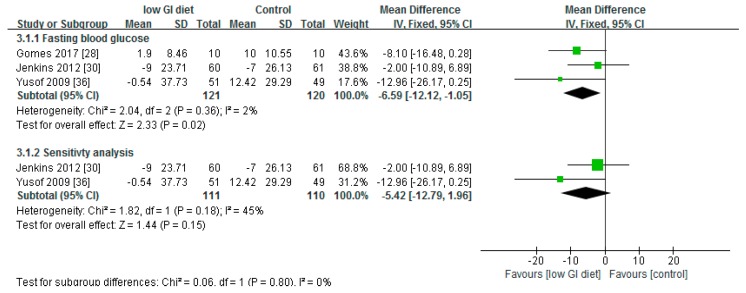
A forest plot showing the effect of low-GI diet on fasting blood glucose (mg/dL).

With respect to evaluating the effect of the low-GI diet on fasting blood glucose compared to the higher-GI diet or control diet, the meta-analysis favoured the low-GI diet. However, while the differences were significant in the meta-analysis (*p* < 0.05), this was not so in the sensitivity test (*p* = 0.15) (removing the study with the most weight) (Figure 5). These sensitivity results (Figure 5) indicate that the effect of the low-GI diet on fasting blood glucose was not very reliable. The fail-safe number (*N*_fs0.05_) with respect to fasting blood glucose was 5. 

Regarding the heterogeneity test, the high *p* value (*p* = 0.36) and the chi-squared statistic (2.04) relative to the degree of freedom (2) would suggest that there was low heterogeneity between the studies. This was confirmed by the results of the *I*^2^ test. The heterogeneity test for fasting blood glucose showed *I*^2^ = 2% for meta-analysis and *I*^2^ = 45% for the sensitivity test, indicating low heterogeneity of the studies included.

## 4. Discussion 

Based on the findings of the systematic review, the low-GI diet resulted in greater improvement in glycated haemoglobin [29,30] and fasting blood glucose [28,29,34,36] compared to higher-GI diet or control in patients with type 2 diabetes. In the study by Yusof et al. [36], although the effect on HbA1c was not significantly different between the low-GI diet and the higher-GI diet or control, the improvement within the low-GI group was more pronounced and of clinical benefit. However, compared with the American Diabetes Association (ADA) diet, the low-GI diet achieved equivalent control of HbA1c using less medication [31] and for patients on diet alone with optimal glycaemic control, long-term HbA1c was not affected by altering the GI [35]. The findings of this systematic review confirm the results of earlier systematic review by Thomas and Elliot [8] which suggests that lowering the glycaemic index of food may improve glycated haemoglobin in patients with diabetes. However, the difference between this review and the Thomas and Elliot [8] study is with respect to the second outcome measure. While fasting glucose was the outcome of interest in this review, it was fructosamine in the review by Thomas and Elliot [8]. Regarding the risk of bias, 100% of the studies showed low risk of selection bias while all the studies demonstrated 100% low risk of bias or unclear risk of bias in relation to blinding, incomplete outcome data, selective reporting, and other potential sources of bias except for one study that showed a high risk of bias with respect to the blinding of participants and personnel.

There was no statistically significant difference between the low-GI and higher-GI diets in relation to HbA1c and fasting blood glucose in the study by Visek et al. [34]. Acute glycaemic control was variable over the 3-day period in the study by Gonçalves Reis and Dullius [32] following the adoption of low-GI diet. While the first day of the study demonstrated a significant difference in acute glycaemic control, differences were not significant on the second or third day [32]. Stenvers et al. [33] found no beneficial effect of the low-glycaemic response liquid meal with respect to fasting blood glucose. The differences observed between these studies in terms of their results may be due to the limitations of using HbA1c in identifying the daily changes in glycaemia [17]. The use of HbA1c may not detect the harmful effects of excessive postprandial hyperglycaemic excursions and the risk of hypoglycaemia [7]. According to Chiu and Taylor [7], the contribution of postprandial glucose may be up to 70% in daily hyperglycaemia, and the contribution of fasting glucose increases as glycaemic control worsens. This may explain why the association between dietary GI and HbA1c is not consistent across studies [7]. Therefore, the use of a meta-analysis of both fasting blood glucose and HbA1c levels to evaluate the effect of dietary GI in patients with diabetes is a useful strategy of eliminating the limitation of using HbA1c to assess GI exposure. Other factors to consider include the nature of the starch, particle size, pH, the amount of fibre, fat, and protein, and the cooking method and time, which may affect the GI of food and its effect on blood glucose response, and lead to differences in outcomes of studies [15]. The differences in the classification of low- and high-GI diets may also have effect on outcomes of studies [37]. 

The Food and Agricultural Organisation [9] has recommended the use of the glycaemic index of foods in clinical applications in patients with diabetes, and that the glycaemic index be used as a useful indicator of the impact of food on the blood glucose response. Recently, the American Diabetes Association (ADA) [38] recommended that patients with diabetes consume carbohydrates from vegetables, fruits, legumes, whole grains, and dairy products. The ADA [38] also recommended that emphasis should be placed on foods which are higher in fibre and lower in glycaemic load as opposed to other sources, especially those with added sugar. The glycaemic load is the product of the glycaemic index of food or diet and the grams of available carbohydrate in that food or diet divided by 100 [9,15]. These recommendations are in line with the findings of the current systematic review and meta-analysis, which showed that low-GI diets were more effective in controlling glycated haemoglobin than higher-GI diets. The position of the ADA [38] with respect to the mixed results of glycaemic index or glycaemic load in patients with diabetes is also true in relation to the findings of the studies included in this review. Foods with low GI have demonstrated beneficial effects on glucose control in short-term trials in patients with type 2 diabetes [13]. However, the higher intake of sucrose or fructose and the long-term use of high-GI diets can place higher metabolic demands on the body in relation to higher insulin requirements [6,39,40]. 

## 5. Strengths and Limitation of the Study

This review used a systematic approach and meta-analysis to provide contemporary evidence on the positive effects of low glycaemic index diet on fasting blood glucose and glycated haemoglobin. The limitation is in the number of studies included in the meta-analysis. In addition, most of the studies had relatively small sample sizes. The availability and inclusion of more studies would have increased its wider application.

## 6. Conclusions

The findings of this systematic review and meta-analysis have shown that low-GI diets are more effective in controlling HbA1c and fasting blood glucose compared with higher-GI diets or control diets in patients with type 2 diabetes. Although the outcomes of the individual studies were sometimes different with respect to the variables of interest, the results of the meta-analysis and sensitivity tests have demonstrated significant differences (*p* < 0.001 and *p* < 0.001, respectively) between low-GI diets and higher-GI diets or control diets in relation to glycated haemoglobin. In addition, differences between the low-GI diet and the higher-GI diet or control were significant (*p* < 0.05) with respect to the fasting blood glucose following meta-analysis. 

## 7. Perspectives for Future Research

It will useful to evaluate the long-term effectiveness of low-glycaemic index diet in patients with type 2 diabetes.

## Figures and Tables

**Table 1:** Search terms and search strategy.

Patient/Population	Intervention	Comparator	Study Designs	Combining Search Terms
Patients with diabetes	Low-glycaemic index diet	Higher-glycaemic index diet or control	Randomised controlled trial	
Patients with diabetes OR type 2 diabetes OR diabetes OR diabetes complications OR diabetes mellitus, type 2 OR diabetes mellitus	Glycaemic index OR glycemic index OR glycaemic load OR glycaemic indices or glycaemic index number or glycaemic index numbers		#1 Randomised controlled trial OR controlled clinical trial OR randomized OR placebo OR drug therapy OR randomly OR trial OR groups #2 “Animals” NOT “Humans” #3 #1 NOT #2	Column 1 and Column 2 and Column 3

**Table 2:** Criteria for considering studies for the review based on the Population, Intervention, Comparator, Outcomes and Study designs (PICOS) structure.

	Inclusion Criteria	Exclusion Criteria
Population	Adult patients (≥18 years) with type 2 diabetes	Studies involving patients with type 1 diabetes or gestational diabetes and animal studies. Studies involving children with diabetes or healthy adults.
Intervention	Low-glycaemic index diet	Studies involving dietary supplements
Comparator	Higher-glycaemic index diet and/or control	Studies involving additional supplements
Outcomes	Blood glucose parameters: Glycated haemoglobin, fasting blood glucose	Qualitative outcomes
Types of study: quantitative	Randomised controlled trials	Observational studies Letters Comments Reviews

**Table 3:** Summary of studies included in the systematic review.

Citation	Length of Study	Study Type	Sample Size	Age (Years)	Diabetes Duration (Years)	Interventions	Glycated Haemoglobin (HbA1c) %	Blood Glucose	Dietary Glycaemic Index
Gomes et al. [28]	1 month	Parallel Design	20	# 42.4 ± 5.1	# Low-GI (Glycaemic Index) diet (4.8 ± 1.5) higher-GI diet (4.9 ± 1.6)	Low-GI diet versus higher-GI diet	No data	*# Baseline Low-GI diet = 148.9 ± 8.2 vs. higher-GI diet 147.8 ± 10.7 30 days Low-GI diet = 150.8 ± 8.7 vs. higher-GI diet = 157.8 ± 10.4 *p* = 0.43	## Baseline Low-GI diet = 63 ± 6 vs. higher-GI diet = 66 ± 4 30 days Low-GI diet = 54 ± 4 vs. higher-GI diet = 72 ± 3 *p* = 0.005
Jenkins et al. [29]	6 months	Parallel Design	210	# Low-GI diet = 60 (10) High-cereal fibre diet = 61 (9)	# Low-GI diet = 8.3 (6.5) High-cereal fibre diet = 7.2 (5.9)	Low-GI diet versus high-cereal fibre diet	Low-GI diet Δ = −0.5% (95% CI, −0.61% to −0.39%) vs. high-cereal fibre diet Δ = −0.18% (95% CI, −0.29% to −0.07%) *p* < 0.001	* (Mean) Week 0 Low-GI diet = 138.8 vs. high-cereal fibre diet = 141.2 Week 24 Low-GI diet = 127.7 vs. high-cereal fibre diet = 136.8 *p* = 0.02	#### Week 0 Low-GI diet = 80.8 (79.6–82.0) vs. high-cereal fibre diet = 81.5 (80.4–82.7) Week 24 Low-GI diet = 69.6 (67.7–71.4) vs. high-cereal fibre diet = 83.5 (82.4–84.7)
Jenkins et al. [30]	3 months	Parallel Design	121	## Low-GI legume diet = 58 (1.3) High-wheat fibre diet = 61 (1.0)	## Low-GI legume diet = 9.2 (8.0) High-wheat fibre diet = 8.6 (0.8)	Low-GI legume diet vs. high-wheat fibre diet	Low GI legume diet Δ = −0.5% (95%, −0.6% to −0.4%) vs. high-wheat fibre diet Δ = −0.3% (95% Cl, −0.4 to −0.2%) *p* < 0.001	*#### Baseline Low-GI legume diet = 141 (135–147) (95% CI) vs. high-wheat fibre diet = 134 (127–141) (95% CI) End of study Low-GI legume diet = 132(126–138) (95% CI) vs. high-wheat fibre diet = 127 (121–133) (95% CI) *p* = 0.001	#### Baseline Low-GI legume diet = 80 (79–82) (95% CI) vs. high-wheat fibre diet = 78 (77–80) (95% CI) End of study Low-GI legume diet = 66 (64–67) (95% CI) vs. high-wheat fibre diet = 82 (81–83) (95% CI) *p* < 0.001
Ma et al. [31]	12 months	Parallel Design	40	# 53.53 ± 8.40	# 9.32 ± 9.66	Low-GI diet vs. American Diabetes Association diet (ADA)	## Baseline Low-GI diet = 8.74 ± 0.29% vs. baseline ADA diet = 8.1 ± 0.28% 12 months Low-GI diet = 8.39 ± 0.30% vs. 12-month ADA diet = 7.67 ± 0.28% *p* = 0.08	No data	## Baseline Low-GI diet = 79.35 ± 1.36 vs. ADA diet = 82.03 ± 1.31 12 months Low-GI diet = 76.64 ± 1.46 vs. ADA diet = 80.36 ± 1.40 *p* = 0.07
Gonçalves Reis and Dullius [32]	2 weeks	Cross-over study	12	# 60 ± 8	# 12 ± 7	Low-GI diet vs. higher-GI diet	No data	*# Low-GI diet first day (127 ± 30) vs. higher-GI diet (148 ± 62) (*p* < 0.05) By the second day FBG levels had the same average value (132 mg/dL) (*p* = 0.78)	No data
Stenvers et al. [33]	22 months	Cross-over study	20	# 60 ± 7	### 5 (1–9)	Low-GR (Glycaemic Response) liquid formula versus free choice (control)	### Baseline Low-GR = 6.5% (6.1–6.9) Control = 6.5% (6.2–6.9) 12 weeks Low-GR = 6.5% (6.3–7.1) Control = 6.6% (6.3–7.0)	**### Baseline Low-GR = 7.3 (6.4–8.1) Control = 6.8 (6.1–7.4) 12 weeks Low-GR = 7.2 (6.5–7.7) Control = 7.0 (6.7–7.8)	No data
Visek et al. [34]	3 months	Cross–over study	20 (12 men + 8 women)	# 62.7 ± 5.8	# 7 ± 4.1	Low-GI diet versus standard diabetic diet	### Low-GI diet = 6.63 (6.08–7.0)% Standard diabetic diet = 6.45 (6.18–6.91)% (*p* > 0.05)	**### Low-GI diet = 6.5 (5.6–8.4) Standard diabetic diet = 6.7 (6.1–7.5) (*p* > 0.05)	### Low-GI diet = 49 (48–51) Standard diabetic diet = 68 (61–72) (*p* < 0.01)
Wolever et al. [35]	12 months	Parallel Design	162	Low-GI diet = 60.6 ± 1.0 Higher-GI diet = 60.4 ± 1.1	No data	Low-GI diet vs. higher-GI diet	## Baseline Low-GI diet = 6.2 ± 0.8% Higher-GI diet = 6.2 ± 1% Outcomes Low-GI diet = 6.34 ± 0.05% Higher-GI diet = 6.34 ± 0.05% *p* > 0.05	No data	## Baseline Low-GI diet = 60.3 ± 0.4 Higher-GI diet = 61.5 ± 0.4 Study Low-GI diet = 55.1 ± 0.4 Higher-GI diet = 63.2 ± 0.4 *p* < 0.001
Yusof et al. [36]	12 weeks	Parallel Design	100	53.5	No data	Low-GI diet vs. conventional carbohydrate exchange (CCE)	## Baseline Low-GI diet = 7.68 ± 1.13% CCE = 7.51 ± 1.24% Week 12 Low-GI diet = 7.2 ± 0.1% CCE = 7.2 ± 0.2% *p* > 0.05	**## Baseline Low-GI diet = 7.33 ± 2.23 CCE = 7.01 ± 1.79 Week 12 Low-GI diet = 7.3 ± 0.3 CCE = 7.7 ± 0.4 *p* < 0.05	# Week 12 Low-GI diet = 57 ± 6 Week 12 CCE = 64 ± 5 *p* < 0.001

Abbreviations: ADA (American Diabetes Association); CCE (conventional carbohydrate exchange); FGB (fasting blood glucose); glycated haemoglobin (HbA1c); GI (glycaemic index); GR (glycaemic response); low-GR (low glycaemic response); Δ (change); * (FBG, mg/dL); ** FBG (mmol/L); # (Mean ± SD); ## (mean ± SEM); ### (Median) (25th–75th percentile); #### (Mean and 95% CI, confidence interval).

## References

[1] Zghebi S.S., Steinke D.T., Carr M.J., Rutter M.K., Emsley R.A., Ashcroft D.M. (2017). Examining trends in type 2 diabetes incidence, prevalence and mortality in the UK between 2004 and 2014. Diabetes Obes. Metab..

[2] Sharma M., Nazareth I., Petersen I. (2016). Trends in incidence, prevalence and prescribing in type 2 diabetes mellitus between 2000 and 2013 in primary care: A retrospective cohort study. BMJ Open.

[3] Haynes-Maslow L., Leone L.A. (2017). Examining the relationship between the food environment and adult diabetes prevalence by county economic and racial composition: An ecological study. BMC Public Health.

[4] Hill J. (2007). Management of diabetes in South Asian communities in the UK. Prim. Health Care.

[5] National Collaborating Centre for Chronic Conditions (NCCCC) (2008). Type 2 Diabetes: National Clinical Guideline for Management in Primary and Secondary Care (Update).

[6] Russell W.R., Baka A., Björck I., Delzenne N., Gao D., Griffiths H.R., Weickert M.O. (2016). Impact of Diet Composition on Blood Glucose Regulation. Crit. Rev. Food Sci. Nutr..

[7] Chiu C., Taylor A. (2011). Dietary hyperglycemia, glycemic index and metabolic retinal diseases. Prog. Retin. Eye Res..

[8] Thomas D.E., Elliott E.J. (2010). The use of low-glycaemic index diets in diabetes control. Br. J. Nutr..

[9] Food and Agricultural Organisation (FAO) (1998). Carbohydrates in Human Nutrition. Report of a Joint FAO/WHO Expert Consultation.

[10] Chang K.T., Lampe J.W., Schwarz Y., Breymeyer K.L., Noar K.A., Song X., Neuhouser M.L. (2012). Low Glycemic Load Experimental Diet More Satiating Than High Glycemic Load Diet. Nutr. Cancer.

[11] Mohan V., Anjana R.M., Gayathri R., Ramya Bai M., Lakshmipriya N., Ruchi V., Sudha V. (2016). Glycemic Index of a Novel High-Fiber White Rice Variety Developed in India—A Randomized Control Trial Study. Diabetes Technol. Ther..

[12] Similä M.E., Valsta L.M., Kontto J.P., Albanes D., Virtamo J. (2011). Low-, medium- and high-glycaemic index carbohydrates and risk of type 2 diabetes in men. Br. J. Nutr..

[13] Jung C., Choi K.M. (2017). Impact of High-Carbohydrate Diet on Metabolic Parameters in Patients with Type 2 Diabetes. Nutrients.

[14] Ikpotokin O.S., Adeleye O.S., Aliu E.D., Osayande A.B. (2017). Ehiabhi, S. Dietary factors in fasting blood glucose levels and weight gain in female Sprague Dawley in rats. J. Clin. Nutr. Diet..

[15] Esfahani A., Wong J.W., Mirrahimi A., Villa C.R., Kendall C.C. (2011). The application of the glycemic index and glycemic load in weight loss: A review of the clinical evidence. IUBMB Life.

[16] Fabricatore A.N., Ebbeling C.B., Wadden T.A., Ludwig D.S. (2011). Continuous glucose monitoring to assess the ecologic validity of dietary glycemic index and glycemic load. Am. J. Clin. Nutr..

[17] Dunning T. (2014). Care of People with Diabetes a Manual of Nursing Practice.

[18] Villegas R., Liu S., Gao Y., Yang G., Li H., Zheng W., Shu X.O. (2007). Prospective study of dietary carbohydrates, glycemic index, glycemic load, and incidence of type 2 diabetes mellitus in middle-aged Chinese women. Arch. Intern. Med..

[19] Krishnan S., Rosenberg L., Singer M., Hu F.B., Djoussé L., Cupples L.A., Palmer J.R. (2007). Glycemic index, glycemic load, and cereal fiber intake and risk of type 2 diabetes in US black women. Arch. Intern. Med..

[20] Ludwig D.S. (2002). The glycemic index: Physiological mechanisms relating to obesity, diabetes, and cardiovascular disease. JAMA.

[21] Sacks F.M., Carey V.J., Anderson C.M., Miller E., Copeland T., Charleston J., Appel L.J. (2014). Effects of high vs low glycemic index of dietary carbohydrate on cardiovascular disease risk factors and insulin sensitivity: The OmniCarb randomized clinical trial. JAMA.

[22] Greenwood D.C., Threapleton D.E., Evans C.L., Cleghorn C.L., Nykjaer C., Woodhead C., Burley V.J. (2013). Glycemic Index, Glycemic Load, Carbohydrates, and Type 2 Diabetes. Diabetes Care.

[23] Fabricatore A.N., Wadden T.A., Ebbeling C.B., Thomas J.G., Stallings V.A., Schwartz S., Ludwig D.S. (2011). Targeting dietary fat or glycemic load in the treatment of obesity and type 2 diabetes: A randomized controlled trial. Diabetes Res. Clin. Pract..

[24] The Nordic Cochrane Centre (2014). Review Manager (RevMan) [Computer Program].

[25] Moher D., Liberati A., Tetzlaff J., Altman D.G., The PRISMA Group (2009). Preferred Reporting Items for Systematic Reviews and Meta-Analyses: The PRISMA Statement. Ann. Intern. Med..

[26] Critical Appraisal Skills Programme (CASP) (2017). Randomised Controlled Trial Checklist. http://docs.wixstatic.com/ugd/dded87_4239299b39f647ca9961f30510f52920.pdf.

[27] Higgins J.P.T., Green S. (2009). Cochrane Handbook for Systematic Reviews of Interventions.

[28] Gomes J.G., Fabrini S.P., Alfenas R.G. (2017). Low glycemic index diet reduces body fat and attenuates inflammatory and metabolic responses in patients with type 2 diabetes. Arch. Endocrinol. Metab..

[29] Jenkins D.A., Kendall C.C., McKeown-Eyssen G., Josse R.G., Silverberg J., Booth G.L., Leiter L.A. (2008). Effect of a low-glycemic index or a high-cereal fiber diet on type 2 diabetes: A randomized trial. JAMA.

[30] Jenkins D.A., Kendall C.C., Augustin L.A., Mitchell S., Sahye-Pudaruth S., Blanco Mejia S., Josse R.G. (2012). Effect of legumes as part of a low glycemic index diet on glycemic control and cardiovascular risk factors in type 2 diabetes mellitus: A randomized controlled trial. Arch. Intern. Med..

[31] Ma Y., Olendzki B.C., Merriam P.A., Chiriboga D.E., Culver A.L., Li W., Pagoto S.L. (2008). A randomized clinical trial comparing low-glycemic index versus ADA dietary education among individuals with type 2 diabetes. Nutrition.

[32] Gonçalves Reis C.E., Dullius J. (2011). Glycemic acute changes in type 2 diabetics caused by low and high glycemic index diets. Nutr. Hosp..

[33] Stenvers D.J., Schouten L.J., Jurgens J., Endert E., Kalsbeek A., Fliers E., Bisschop P.H. (2014). Breakfast replacement with a low-glycaemic response liquid formula in patients with type 2 diabetes: A randomised clinical trial. Br. J. Nutr..

[34] Visek J., Lacigova S., Cechurova D., Rusavy Z. (2014). Comparison of a Low-Glycemic Index vs. Standard Diabetic Diet.

[35] Wolever T., Gibbs A., Mehling C., Chiasson J., Connelly P., Josse R., Ryan E. (2008). The Canadian Trial of Carbohydrates in Diabetes (CCD), a 1-y controlled trial of low-glycemic-index dietary carbohydrate in type 2 diabetes: No effect on glycated hemoglobin but reduction in C-reactive protein. Am. J. Clin. Nutr..

[36] Yusof B.M., Talib R.A., Kamaruddin N.A., Karim N.A., Chinna K., Gilbertson H. (2009). A low-GI diet is associated with a short-term improvement of glycaemic control in Asian patients with type 2 diabetes. Diabetes Obes. Metab..

[37] Miller C.K., Headings A., Peyrot M., Nagaraja H. (2011). A behavioural intervention incorporating specific glycaemic index goals improves dietary quality, weight control and glycaemic control in adults with type 2 diabetes. Public Health Nutr..

[38] American Diabetes Association (2018). Life style management: Standards of Medical Care in Diabetes–2018. Diabetes Care.

[39] Ojo O., Brooke J. (2014). Evaluation of the role of enteral nutrition in managing patients with diabetes: A systematic review. Nutrients.

[40] Widanagamage R.D., Ekanayake S., Welihinda J. (2009). Carbohydrate-rich foods: Glycaemic indices and the effect of constituent macronutrients. Int. J. Food Sci. Nutr..

